# Supplementary material for: Factors Associated with Reduced Heart Rate Variability in the General Japanese Population: The Iwaki Cross-Sectional Research Study
Source: Healthcare (Basel). 2022 Apr 24;10(5):793. doi: 10.3390/healthcare10050793 (PMC9141757; doi:10.3390/healthcare10050793)
Supplement: Supplementary file 1 [file healthcare-10-00793-s001.zip › healthcare-1654942-SI.pdf]

Table S1. Univariate analysis of the association with LH, HF and LF/HF

Characteristics	Unit	LF (ms^2)			HF (ms^2)			LF/HF		
		β	95% CI	p-value	β	95% CI	p-value	β	95% CI	p-value
Age	years	-9.61	-11.69 ~ -7.53	<0.001	-4.85	-6.25 ~ -3.45	<0.001	-0.015	-0.034 ~ 0.004	0.133
Sex	women	-137.86	-203.30 ~ -72.42	<0.001	11.91	-31.75 ~ 55.58	0.593	-0.993	-1.573 ~ -0.412	0.001
BMI	kg/m^2	-9.98	-18.92 ~ -1.04	0.029	-11.27	-17.16 ~ -5.39	<0.001	0.124	0.045 ~ 0.202	0.002
HbA1c	%	-100.07	-152.86 ~ -47.29	<0.001	-71.03	-105.92 ~ -36.14	<0.001	0.166	-0.304 ~ 0.637	0.487
Glycoalbumin	%	-28.94	-45.40 ~ -12.49	0.001	-16.61	-27.51 ~ -5.70	0.003	-0.077	-0.224 ~ 0.069	0.302
Blood glucose	mg/dL	-3.96	-5.94 ~ -1.99	<0.001	-3.36	-4.66 ~ -2.06	<0.001	0.011	-0.006 ~ 0.029	0.215
Triglyceride	mg/dL	-0.20	-0.59 ~ 0.19	0.306	-0.43	-0.69 ~ -0.17	0.001	0.003	0.000 ~ 0.007	0.046
Total cholesterol	mg/dL	-1.46	-2.39 ~ -0.53	0.002	-0.93	-1.55 ~ -0.31	0.003	0.000	-0.008 ~ 0.008	0.958
HDL cholesterol	mg/dL	-1.82	-3.77 ~ 0.13	0.068	0.30	-1.00 ~ 1.59	0.654	-0.023	-0.040 ~ -0.005	0.010
LDL cholesterol	mg/dL	-1.17	-2.25 ~ -0.09	0.033	-0.83	-1.54 ~ -0.12	0.022	0.003	-0.006 ~ 0.013	0.488
ALT	U/L	0.56	-1.73 ~ 2.86	0.629	-1.14	-2.66 ~ 0.38	0.140	0.024	0.003 ~ 0.044	0.023
AST	U/L	-2.95	-7.04 ~ 1.14	0.157	-2.75	-5.45 ~ -0.05	0.046	0.020	-0.016 ~ 0.057	0.267
γ-GTP	U/L	-0.11	-0.90 ~ 0.69	0.796	-0.61	-1.13 ~ -0.08	0.023	0.010	0.003 ~ 0.017	0.007
Creatinine	mg/dL	-6.70	-67.00 ~ 53.60	0.827	-26.06	-65.93 ~ 13.80	0.200	0.059	-0.474 ~ 0.593	0.828
Urea nitrogen	mg/dL	-13.07	-20.24 ~ -5.89	<0.001	-8.11	-12.86 ~ -3.36	0.001	-0.016	-0.080 ~ 0.047	0.614
Plasma pentosidine	pmol/mL	-3.64	-5.60 ~ -1.69	<0.001	-1.45	-2.75 ~ -0.15	0.029	-0.016	-0.033 ~ 0.002	0.080
SBP	mmHg	-3.81	-5.72 ~ -1.90	<0.001	-2.47	-3.73 ~ -1.20	<0.001	-0.004	-0.022 ~ 0.013	0.607
DBP	mmHg	-5.11	-7.96 ~ -2.26	<0.001	-4.85	-6.72 ~ -2.98	<0.001	0.009	-0.016 ~ 0.034	0.482

γ-GTP, γ-glutamyl transferase; ALT, alanine transaminase; AST, aspartate transaminase; CI, confidence interval; DBP, diastolic blood pressure; HbA1c, hemoglobin A1c; HDL, high density lipoprotein; HF, high-frequency component power; LDL, low density lipoprotein; LF, low-frequency component power; SBP, systolic blood pressure.

Table S2. Multivariate analysis of the association with LH, HF and LF/HF (Model 1)

Characteristics	Unit	LF (ms^2)			HF (ms^2)			LF/HF		
		β	95% CI	p-value	β	95% CI	p-value	β	95% CI	p-value
HbA1c	%	-26.87	-81.71 ~ 27.96	0.336	-26.75	-63.91 ~ 10.40	0.158	0.079	-0.427 ~ 0.586	0.758
Glycoalbumin	%	-5.46	-22.11 ~ 11.20	0.520	-7.33	-18.61 ~ 3.95	0.203	-0.011	-0.165 ~ 0.143	0.890
Blood glucose	mg/dL	-1.57	-3.69 ~ 0.55	0.146	-1.68	-3.11 ~ -0.24	0.022	0.005	-0.014 ~ 0.025	0.600
Triglyceride	mg/dL	-0.31	-0.70 ~ 0.09	0.129	-0.34	-0.61 ~ -0.07	0.013	0.001	-0.003 ~ 0.005	0.549
Total cholesterol	mg/dL	-0.30	-1.22 ~ 0.63	0.529	-0.38	-1.01 ~ 0.24	0.230	0.001	-0.007 ~ 0.010	0.753
HDL cholesterol	mg/dL	-0.80	-2.91 ~ 1.32	0.460	-0.25	-1.68 ~ 1.19	0.737	-0.006	-0.026 ~ 0.014	0.548
LDL cholesterol	mg/dL	0.00	-1.07 ~ 1.07	0.999	-0.14	-0.87 ~ 0.58	0.695	0.002	-0.008 ~ 0.012	0.645
ALT	U/L	-0.55	-3.06 ~ 1.95	0.665	-0.44	-2.14 ~ 1.26	0.609	0.003	-0.020 ~ 0.026	0.809
AST	U/L	-0.41	-4.55 ~ 3.73	0.847	-0.06	-2.87 ~ 2.75	0.966	0.004	-0.034 ~ 0.043	0.818
γ-GTP	U/L	-0.44	-1.24 ~ 0.36	0.283	-0.46	-1.00 ~ 0.09	0.098	0.006	-0.001 ~ 0.013	0.117
Creatinine	mg/dL	-23.52	-82.21 ~ 35.17	0.432	-18.65	-58.44 ~ 21.13	0.358	-0.137	-0.679 ~ 0.405	0.620
Urea nitrogen	mg/dL	-2.95	-10.53 ~ 4.63	0.445	-1.70	-6.84 ~ 3.44	0.516	-0.017	-0.087 ~ 0.053	0.634
Plasma pentosidine	pmol/mL	-1.64	-3.59 ~ 0.32	0.101	-0.53	-1.86 ~ 0.80	0.433	-0.010	-0.028 ~ 0.008	0.282
SBP	mmHg	-1.15	-3.18 ~ 0.88	0.265	-0.61	-1.99 ~ 0.76	0.382	-0.011	-0.029 ~ 0.008	0.264
DBP	mmHg	-3.54	-6.44 ~ -0.63	0.017	-3.28	-5.24 ~ -1.32	0.001	-0.003	-0.030 ~ 0.024	0.823

Model 1: Adjusted for age, sex, and BMI.

γ-GTP, γ-glutamyl transferase; ALT, alanine transaminase; AST, aspartate transaminase; CI, confidence interval; DBP, diastolic blood pressure; HbA1c, hemoglobin A1c; HDL, high density lipoprotein; HF, high-frequency component power; LDL, low density lipoprotein; LF, low-frequency component power; SBP, systolic blood pressure.

Table S3. Multivariate analysis of the association with LH, HF and LF/HF (Model 2)

		LF (ms^2)			HF (ms^2)			LF/HF		
Characteristics	Unit	β	95% CI	p-value	β	95% CI	p-value	β	95% CI	p-value
HbA1c	%	-22.15	-78.47 ~ 34.17	0.440	-26.01	-63.73 ~ 11.70	0.176	0.188	-0.330 ~ 0.706	0.476
Glycoalbumin	%	-6.00	-22.97 ~ 10.98	0.488	-7.14	-18.51 ~ 4.23	0.218	0.005	-0.152 ~ 0.161	0.954
Blood glucose	mg/dL	-1.50	-3.69 ~ 0.68	0.177	-1.82	-3.28 ~ -0.36	0.014	0.006	-0.014 ~ 0.026	0.542
Triglyceride	mg/dL	-0.25	-0.66 ~ 0.15	0.223	-0.34	-0.61 ~ -0.07	0.015	0.001	-0.002 ~ 0.005	0.484
Total cholesterol	mg/dL	-0.23	-1.18 ~ 0.71	0.626	-0.28	-0.92 ~ 0.35	0.378	0.001	-0.008 ~ 0.009	0.892
HDL cholesterol	mg/dL	-0.84	-3.07 ~ 1.38	0.457	-0.34	-1.83 ~ 1.15	0.657	-0.010	-0.030 ~ 0.010	0.335
LDL cholesterol	mg/dL	0.01	-1.10 ~ 1.12	0.984	0.00	-0.74 ~ 0.74	0.997	0.002	-0.008 ~ 0.012	0.720
ALT	U/L	-0.58	-3.14 ~ 1.97	0.654	-0.35	-2.07 ~ 1.36	0.685	0.005	-0.019 ~ 0.028	0.694
AST	U/L	-0.58	-4.82 ~ 3.67	0.790	-0.16	-3.00 ~ 2.69	0.914	0.005	-0.034 ~ 0.044	0.815
γ-GTP	U/L	-0.34	-1.16 ~ 0.49	0.428	-0.48	-1.04 ~ 0.07	0.088	0.006	-0.002 ~ 0.014	0.125
Creatinine	mg/dL	-22.11	-82.14 ~ 37.92	0.470	-22.53	-62.73 ~ 17.67	0.272	-0.078	-0.630 ~ 0.475	0.783
Urea nitrogen	mg/dL	-4.73	-12.78 ~ 3.31	0.249	-2.56	-7.95 ~ 2.84	0.353	-0.012	-0.087 ~ 0.062	0.741
Plasma pentosidine	pmol/mL	-1.91	-3.99 ~ 0.17	0.072	-0.54	-1.93 ~ 0.86	0.450	-0.009	-0.028 ~ 0.010	0.346
SBP	mmHg	-1.09	-3.18 ~ 1.01	0.309	-0.66	-2.07 ~ 0.74	0.355	-0.011	-0.031 ~ 0.008	0.248
DBP	mmHg	-3.45	-6.45 ~ -0.44	0.025	-3.39	-5.40 ~ -1.38	0.001	-0.007	-0.034 ~ 0.021	0.642

Model 2: Adjusted for age, sex, BMI, antihypertensive use, physical activity (non-winter and winter seasons), smoking, and alcohol consumption.

γ-GTP, γ-glutamyl transferase; ALT, alanine transaminase; AST, aspartate transaminase; CI, confidence interval; DBP, diastolic blood pressure; HbA1c, hemoglobin A1c; HDL, high density lipoprotein; HF, high-frequency component power; LDL, low density lipoprotein; LF, low-frequency component power; SBP, systolic blood pressure.
